# Knowledge and practice of traditional healers in oral health in the Bui Division, Cameroon

**DOI:** 10.1186/1746-4269-7-6

**Published:** 2011-01-15

**Authors:** Ashu M Agbor, Sudeshni Naidoo

**Affiliations:** 1 Department of Community Dentistry, University of the Western Cape, Tygerberg, South Africa

## Abstract

**Background:**

The majority of Cameroonians depend on traditional medicines for their health care needs and about seven per cent of the average household health budget is spent on traditional medicines irrespective of their incomes. The aim of the present study was to determine the oral care knowledge and practices of Traditional Healers (TH) on oral health delivery in the urban and rural areas of Bui Division of Cameroon and the objectives to determine the cost of treatment and reasons why people visit TH.

**Methods:**

The present study was cross sectional and utilized semi-structured questionnaires to collect data.

**Results:**

The sample consisted of 21 TH and 52 clients of TH. Sixty two percent of the TH's were above 40 years and 90% male. The mean age was 46 years (range 20-77 years). Twenty four percent of the TH practiced as herbalists and the remainder both divination and herbalism. Sixty seven percent of people in the Bui Division, who patronize TH for their oral health needs, fall within the 20-40 year age group. There is little collaboration between the oral health workers and TH and only 6% of all patients seen by TH are referred to the dentist. Socio-cultural and economic factors affect the oral health care seeking behavior of patients in this area and only 6.5% of patients visit dental clinics. Reasons for not attending dental clinics included high cost, poor accessibility, superstition and fear. TH's are not experienced in the treatment of pulpitis - the majority of patients who presented with toothache had temporary or no relief, but despite this 67% reported being satisfied with their treatment. Sixty nine percent of the patients visited TH because of low cost - the average cost of treatment with TH (approximately $5) is very low, as compared to conventional treatment ($50).

**Conclusions:**

Traditional healers are willing to co-operate with oral health workers in improving oral health. Since they have a vital role to play in health care seeking attitudes in this community and barriers affecting the oral health seeking behaviours should be removed. Mutual cooperation, collaboration and by integrating TH into primary oral health care services needs to be increased.

## Background

A traditional healer is a person who has no formal medical training, but is recognized by the community in which he/she lives as being competent to provide health care by using plant, animal and mineral substances and certain other methods based on social, cultural and religious background as well as the knowledge, attitudes and beliefs that are prevalent in the community regarding physical, mental and social well-being and the causation of the disease and disability [1]. According to the World Heath Organization (WHO), more than 80 percent of Africans rely on traditional medicine and indigenous knowledge to meet their health needs [1]. This is due to the fact that traditional medicine is accessible, affordable, culturally and socially acceptable and most people prefer it to the 'exorbitantly priced' conventional Western medicine. With the legalization of traditional medicine as a complimentary health care service to primary health care in Cameroon, the role of the traditional healer will be vital in the promotion of health especially in resource poor settings and rural areas where they may be the only source of health care [2].

Since colonial times, Western medicine was the only formally accepted medicine in Cameroon. Traditional medicinal practices were condemned as witchcraft or sorcery and discouraged. Despite this, the practice of traditional medicine has survived clandestinely in Cameroon. One of the main reasons Cameroonians still favor traditional medicines is financial - they resort to traditional medicine because they cannot afford pharmaceutical medicaments or conventional medical care [3].

The oral health care work force in Cameroon consists of 220 dentists all trained abroad. Nearly all are located in the two big cities of Douala and Yaoundé serving just 20% of the country's population. On the other hand, it is estimated that there are more than 20 000 traditional healers (TH) in the country serving both the rural and urban population. Rural populations do not have access to the services of trained oral health personnel due to cost constraints and poor accessibility. Today, seven per cent of the average household health budget is spent on traditional medicines. Nearly twice as many people from poor households rely on traditional medicine as do people from rich households [4]. The recognition and integration of traditional medicine into the health system of Cameroon was officially proposed in 1981. Since then, traditional medicine has been recognized, but not regulated by the Ministry of Health. In 1995, a presidential decree no. 95-040 of July 3, 1995 gave TH in Cameroon, the authorization to create associations at both provincial and national levels to manage their activities [5].

In recognition of the fact that traditional medicine is "the most affordable and accessible system of health care for the majority of the African rural population," the Organization for African Unity (now the African Union) declared the years 2001-2010 to be the 'Decade for African Traditional Medicine'. The aim of this declaration was to bring together all the stakeholders in health care in an effort to make traditional medicine "safe, efficacious, affordable and available to the vast majority of African people" [6]. One of the tasks of the WHO (Africa Region) is to assist countries in ensuring that the African population enjoys improved levels of oral health and function through a significant reduction of all oral diseases and conditions that are prevalent in the region, with equitable access to cost-effective quality oral health care and adoption of healthy lifestyles.

Despite much research in recent times regarding health (medical) sector collaboration with TH, there is paucity of literature with regard to the role of TH in the provision of oral health care and in the diagnosis and management of the common oral problems including the oral manifestations of HIV/AIDS. Given shrinking health budgets, economic constraints and the diminishing capacity for oral health personnel to handle the burden of oral diseases throughout much of Sub-Saharan Africa, it would seem logical to develop and enhance co-operation and collaboration between the formal oral health services and TH to bring available resources in the health sector to serve the population for better oral health and HIV/AIDS prevention.

Traditional healers are considered to be effective agents of change as they command authority in their communities, function as psychologists, marriage and family counselors, physicians and legal and political advisors [7]. They are also the legitimate interpreters of customary rules of conduct, morality and values. TH provide client-centered, personalized health care that is culturally appropriate and tailored to meet the needs and expectations of the client paying special respect to social and spiritual matters [8].

Lewis et al. [9] reported on the oral health care knowledge and practices of African TH from two communities: Zonkizizwe and Dube in the Gauteng Province, South Africa. According to their findings, more than 90% of TH from both areas correctly identified photographs of gingival inflammation, dental caries and oral candidiasis. More than half reported patients presented with mouth problems such as toothache, swollen gums and oral candidiasis. Considering that oral candidiasis has been reported as the most prevalent oral manifestation of HIV/AIDS and the fact that almost all TH can recognize oral candidiasis suggests that TH could play an essential role in the efforts to address early diagnosis of the oral manifestations of HIV/AIDS [7,9].

A study carried out in Nigeria [10] found that TH were providing dental care, but their work was not integrated with that of a dentist. He reported that while TH were open to collaborating with dental professionals, the reverse was not true. TH are more numerous than dental and medical practitioners and are widely accepted by a large proportion of the population, therefore it is logical that their work be integrated with that of dental and medical practitioners. In Africa and some parts of Asia chewing sticks are used for plaque removal [11,12]. Most plants used as chewing sticks contain fluoride and/or have antimicrobial, anti-cariogenic or anti-inflammatory properties [12,13].

Ngilisho et al. [14] reported that sixty per cent of the villagers in Tanga region, Tanzania who suffered from toothache sought treatment from TH. They were treated with local herbs and obtained pain relief for more than six months. The authors concluded that the presence of modern health facilities did not influence the villagers' use of TH. Hence, it could be surmised that TH play an important role in the relief of acute pain, in underserved rural areas.

Therapeutic methods used by African TH include psychosocial counseling, simple surgical procedures, rituals and symbolism [15]. The types of medications used by TH can be classified as preventive and prophylactic medications [16], treatment for ailments [17] and medications used to "destroy the power in others" [18-22]. The need to identify and recognize the beneficial effects of traditionally used plants and medicaments has been recognized [19].

Hamza et al. [18] investigated the antifungal activity of traditionally used Tanzanian plants and found good correlation between traditional therapeutic use and *in vitro *antifungal activity and corroborated the importance of ethnobotanical surveys for screening plants as a potential source for bioactive components that may have preventive, prophylactic or treatment properties for oral and other diseases. Sarita and Tuominen [23] investigating the pattern of utilization of medical and dental health care services in rural Tanzania reported that indigenous home remedies were the only treatments used for managing dental problems, while for medical problems a TH was the most commonly used. Since the pattern of utilization of health care services differed for medical and dental problems, it should be taken into account when planning comprehensive health care services for rural African societies.

However, one needs to be aware that some traditional practices may be harmful for example, the practice of extracting tooth buds and of rubbing herbs on to the gingivae of children to treat fevers and diarrhea, as has been documented in countries such as Tanzania and Uganda [14]. There is a need for health education programmes [14]. Discouraging the adoption of deeply rooted traditional practices that are potentially hazardous to health and oral health needs to be made a public health priority [15]. This could be achieved by educating not only the general public, but also the TH and community leaders that convey the knowledge to their people.

There have been many instances where TH have collaborated with the health sector. Wilkinson et al. [24] investigated the potential for TH to act as tuberculosis (TB) treatment supervisors. Although only four per cent of the study population believed that TH could cure TB, 84% stated that they would consider choosing a healer as a treatment supervisor. Eighty eight per cent of healers reported having referred patients with suspected TB to hospitals for treatment and all the healers were keen to collaborate with health services and to act as treatment supervisors.

In an earlier report investigating the relationship between traditional and modern medicine Edwards [25] found that while traditional and modern practitioners worked from different theoretical orientations, they were in significant agreement as to both diagnosis and treatment of patients when faced with the same limited choice of options. Furthermore, patients perceived both the traditional and modern practitioners as being more or less equally helpful.

In Uganda, THETA (Traditional and modern health practitioners together against AIDS and other diseases), is promoting collaboration between traditional and biomedical health workers in the prevention and care of sexually transmitted infections (STIs) including HIV/AIDS. Projects involve collaboration in clinical trials to study the effectiveness of herbal treatments for opportunistic infections and to empower traditional medicine practitioners to offer counseling and education on STIs/AIDS.

A study by Homsy and King [26] concluded that traditional healers could be trained as counselors and educators to disseminate HIV/AIDS information and prevention practices between their peers and communities. Case studies indicate that TH are capable of performing at least as well as their biomedical counterparts as AIDS educators and counselors. Of concern to Homsy and King [26] however, was the failure of many projects to provide systematic follow-up to healers after their initial training. Such follow-up is essential to support healers in dealing with unfamiliar issues such as condom use and death and dying. Masauso Nzima et al. [27] carried out a similar study in four Copperbelt towns in Zambia whereby TH received AIDS training and how to counsel clients on safe sex behaviors, together with follow-up monthly meetings.

A qualitative investigation by Abdool Karim [28] exploring potential preventative health roles that TH could play with regard to HIV prevention, recommended that TH be incorporated into AIDS prevention programmes where they can play a role in community-based AIDS education. There is increasing recognition on the role of TH in preventing and controlling HIV/AIDS and other sexually transmitted infections (STIs) [29].

Green [29] made some important recommendations for one to consider when planning collaborative work with TH:

• Be fair and democratic in selecting healers for training

• Try to identify and train motivated healers who are respected in their communities

• Do not make membership of a TH's association a requirement for participation in HIV/AIDS training

• Encourage healers to promote sexual abstinence among youth, and fidelity within marriage among adults.

The World Health Organization (WHO) and other official groups have acknowledge the potential effectiveness of TH as primary caregivers and the potential efficacy of their treatments in the fight against HIV and AIDS, sexually transmitted disease, and other infectious diseases [30]. The WHO also supports the integration of Western medicine and traditional healing and encourages referrals between the two groups. In South Africa, TH have their own organization (Board of TH) that is recognized by the Department of Health and by the Ministry of Health respectively. Among the Zulu population, TH serve many functions in the community, such as the role of a minister of religion, legal advisor, healer, custodian of history and tradition and community organizer [17,31,32].

Traditional healing has always been a component of health care in Cameroon but the actual contribution of TH to oral health care in the Bui Division of Cameroon is not known.

The aim of the present study was to assess the knowledge and practices of TH and determine the extent to which TH can diagnose oral conditions and how they can be incorporated into oral health care and prevention of oral diseases.

## Methods

This is a cross-sectional study design in rural and urban populations of Bui Division, in the North West Region of Cameroon. In Bui Division, general and oral care health delivery is carried out by 9 medical doctors and 1 dentist serving a population of 133 000 people. There are more than 500 TH registered with the TH's association and treat common problems like diarrohea and malaria to more complex cases involving bone setting and serving as traditional birth attendants.

Inhabitants residing in rural and urban areas of Bui division who sought treatment in hospital or from TH's for oral problems and individuals practicing as TH in the Bui divisions were interviewed. TH were randomly chosen from each of the sub-clans in the 3 towns located in Bui division. Bui division is one of the five divisions in the North West Region of Cameroon. Kumbo, it's headquarters is the second largest town in the region and is located 110 km from the regional capital, Bamenda. The area is mountainous with poor road networks and basic infrastructure and the majority of inhabitants are subsistent farmers. The subclans division is based on 3 linguistic communities - the Nso, Oku and Noni whose headquarters are based at the towns of Kumbo, Oku and Nkor Noni respectively.

A workshop was held to determine the knowledge and practices of TH on oral diseases and oral health care. The objectives were to determine their knowledge of the common oral diseases, the type of treatments administered to treat oral problems and the cost of TH's care as compared to conventional dental treatment provided in local dental clinics. Colour photographs of common oral diseases and oral lesions were used for pre-testing of the knowledge of TH, a short training workshop was carried out followed by post-testing 3 months after the initial training workshop.

Questionnaires were administered to a convenience sample of inhabitants who used the services of THs. TH questionnaires elicited data regarding their oral health knowledge and willingness to screen and educate their clients about oral health care. Closed and open ended questionnaires were used for both groups. Informed consent was obtained from all the participants.

Data were categorized, coded and then entered into the computer. The data was captured in Excel. Basic descriptive analysis was done using the Excel environment. The database was imported into SPSS^® ^to perform complex statistical analyses. Descriptive statistics were used to describe the demographic factors. The independent t-test was used to determine correlation between the scale variables. The Chi-square test was used to determine the association between the nominal and the ordinal variables.

## Results

A total of 21 TH and 52 clients participated in the study. The ages of the TH ranged from 20 to 77 years with the average age of 46.09 years and a standard deviation of 14.29. Sixty two percent of the TH was over 40 years and 90% were male. More than a fifth (23.8%) of the TH were herbalists and the remainder practiced both divination and herbal medicine. Of the 52 clients, slightly more than half were males. The age distributions can be seen in Table 1.

**Table 1:** Age distribution of TH and their clients

Age range (years)	TH N(%)	Clients N(%)
< 20 yrs	1 (4.6)	8 (15.4)
21-30 yrs	1 (4.6)	22 (42.3)
31-40 yrs	6 (28.6)	13 (25.0)
>40 years	13 (61.9)	9 (17.3)
Total	21(100)	52(100)

### (a) Traditional healers

**Figure 1:**
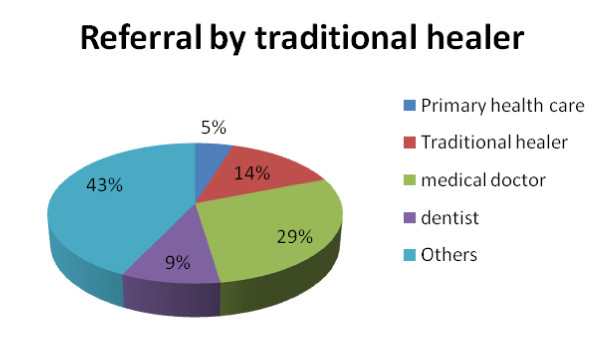
**Referral by TH**.

Seventy one percent of the TH had a primary and high school education, but had no formal training for their profession. More than two thirds (76.2%) were registered with the TH's association and the average duration of training of TH was 7.5 years (range 4 to 10 years). The average duration of practice of TH was 21 years (range 2 to 41 years). Three quarter of the TH were about half an hour away from the nearest health facility. All TH reported that if necessary, they referred patients to other health care workers (Figure 1).

Seventy one percent of TH reported that they knew the cause of HIV/AIDS and 67% stated that they could treat oral HIV lesions. Some of the causes of AIDS/HIV reported by TH included unprotected sex, contaminated needles and contact with blood. All TH were able to accurately detect candidiasis, dental caries and gum diseases from photographs after a training workshop. There was a significant increase in knowledge in the diagnosis of aphthous ulcers, tongue cancer, and Kaposi sarcoma (Table 2).

**Table 2:** Identification of common oral conditions from photographs

	Lesion/Disease	Pre-test correct	Post-test correct	Percentage difference	p value
A	Caries	21 (100)	21 (100)	0.00	1
B	Gum Disease	11 (53.33)	21 (100)	46.67	< 0.001
C	Tongue Cancer	2 (9.52)	18 (85.68)	76.16	0.015
D	Kaposi Sarcoma	4 (19)	17 (80.92)	61.92	0.003
E	Aphthous Ulcers	2 (9.52)	19 (90.44)	80.92	0.004
F	Candida	17 (80.92)	21 (100)	19.08	0.036
**MEAN**		**10(45.38)**	**20(92.84)**	**79.87**	**0.004**

There was a statistical significant increase (p = 0.004) in knowledge from pre-test to post-test for all the six diseases.

Tables 3 shows the treatment and materials used for oral lesions. The barks of trees, herbs and roots are used to make mouth washes. Their reported benefits include pain relieve and softening tooth before extractions

**Table 3:** Type of treatment given by TH

Type of treatment provided	Percentage
Mouth wash from bark of tree extracts	67.0
Pain relieving herbs	11.0
Fume inhalations(from burnt spices in palm oil) that remove worms from infected tooth	11.0
Softens tooth before extractions	5.5
Application of powder from bark of tree to stop pain	5.5

### (b) Clients

Fifty two participants who reported having oral problems and had dental treatment were interviewed. The age of participants ranged from 9 to 70 years; SD = 0.503(Table 1). More than half live below $100 per month. Most patients reported visiting the TH because of the low cost (69%), TH understand their problems better (12%), fear of death from tooth extractions (8%), and hospital or clinics were too far away (5%). More than two thirds (67.3%) were satisfied with the treatment the TH provided and those who were dissatisfied reported that there was no significant change in their presented complaint and pain persisted even after treatment. Table 4 shows the proximity of TH and health facilities. For 76% it takes about 30 minutes to get to a TH and for more than half an average of 4 hours to get to the nearest oral health care facility.

**Table 4:** Proximity of patients to TH and health facilities

Time	Distance of patient from TH N (%)	Distance from oral health facility N (%)
< 30 min	38(76)	2(3.9)
30 min - 1 hr	7(14)	17(33.4)
1 hr - 4 hrs	4(8)	5(9.8)
>4 hrs	1(2)	27(52.9)

## Discussion

### (a) Traditional healers

The fact that more than two thirds of TH in the present study were older than 40 years implies that if speedy interventions to incorporate them into oral health education and promotion efforts are not made, the legacy of the use of traditional medicines in the treatment of oral lesions may be lost [33]. The most senior TH were illiterate, but form the majority of the trainers and are respected community leaders; therefore there is the existence of knowledge transfer from the elderly to younger healers. The higher preponderance of males in the TH sample is because traditional medicine is a male-dominated profession in the Bui division concurring with the findings of Gessler et al. [34].

Less than half of the TH had formal training. Furthermore, the training was not standardized, as most were trained by fathers, uncles and other senior TH. The average duration of training for an herbalist was 7.5 years with training ranging from 4 to 10 years depending on the ability of the apprentice. It can be concluded that because of their longer training, herbalists have good knowledge and skills to treat patients, unlike diviners who do not undergo any form of apprenticeship.

Most of the TH in the Bui division diagnose and treat problems. This finding is similar to studies carried out in the Tanzania and Zambia [2,35]. Of the 21 TH in this study, only 76% of them are registered with the Traditional Healer's Association of Cameroon. Increasing the number of registered TH may be a way of regulating the profession and controlling bad practices.

Despite the fact that more than two thirds of TH are close to basic primary health facilities, most of their referral for oral problems are to medical doctors and other TH. Only few patients were referred to the dental clinic. Most patients visit TH first, before a medical or dental practitioner [36-39]. Some encourage the use of self medication including herbs and common analgesics because of the perception that referral to a dental clinic will be very expensive.

Seventy one percent of TH reported that they were aware of the causes of HIV/AIDS and this is comparable to the findings of Pelzer and colleagues [36]. TH need to be educated on other practices that can transmit the virus and should also be trained in HIV counseling, condom distribution, community HIV/AIDS and STI education. Sixty seven percent of TH reported that they treat patients for mouth problems. With regard to HIV infections, many TH report that their treatments help patients to regain their appetite, cure the opportunistic lesions and increase the "red blood cell count". Their perceived impression is, that if you can increase the red blood cells, the patient feels better [2,3,33,35,36].

Pre-testing the TH for knowledge of oral lesions revealed that they could diagnose candidiasis, dental caries and gum diseases from photographs of 6 oral lesions. Similar findings had been found by Lewis et al. [6] and Rudolph et al. [8] in South Africa. All the TH reported that they would be willing to learn more about common mouth problems in adult and children and were willing to screen patients for oral manifestations of HIV. Furthermore, they were also interested in educating their clients about oral health care if given adequate training. A study carried out in Yaoundé, Cameroon has long-established that although the education level varies widely, traditional practitioners have a deep thirst for knowledge and yearn for greater inclusion into the public health sector [3]. This is supported by other studies from Africa [2,33,35,36].

In the present study, treatment for oral disease by TH included the use of mouth washes, pain relieving herbs, fumes from burnt food spices used to remove "worms" from infected teeth and the application of powder made from bark of trees to stop pain. The use of natural products for the treatment of other oral disease like herpes zoster, tuberculosis, candidiasis and toothache have also been reported by Kisangau [33] and Tapsoba and Deschamps [19].

However, one of the problems associated with the use of herbal treatments is that some of them have never been rigorously evaluated or standardized (in terms of a standard pharmacopeia) [2]. They are often poorly packed and preserved, limiting their usefulness, accessibility and shelf life. Hillenbrand [3] reported that all healers interviewed acknowledged that the plants that they use can be toxic as some of their ingredients can have profound effects on the mouth, stomach, and the entire gastro-intestinal tract with severe consequences like "iatrogenic gastritis and colitis". A common complaint about traditional medicine is that healers claim they can treat everything whether they have a sound knowledge of the aetiology or patho-physiology of the disease or not. This calls for caution about the efficacy of their treatment modalities.

### (b) Clients

Two thirds of clients in the present study were aged between 21-40 years and this could be due not only to the small sample size, but also the demographic composition of the area since in Cameroon a larger proportion of the population is younger. Maclean and Bannerman [36] found that a higher number of older people visited TH than the younger age groups.

Kayombo and colleagues [2] have reported that the involvement of oral health into the primary health care system is still minimal. The average cost of treatment from TH is very low (approximately $5) as compared to conventional treatment ($50). Pelzer et al. [36] suggested that patients visit TH because they provide client-centered and personalized health care that is tailored to meet their needs and expectations, paying special respect to social and spiritual matters. Nevertheless 32% were not satisfied by the treatment provided by the TH, mainly because the pain persisted despite the traditional remedies provided. Treatment for pulpitis was often short-lived.

Reasons for not taking up oral health services included poor accessibility to oral health facilities and unaffordable private dental care. Furthermore, people were afraid of dental clinics and preferred cheap palliative treatments instead [40]. There was also a lack of knowledge of the role of the dental team as efficient oral health care providers as there is an acute shortage of oral health care workers (only one dentist, 2 dental therapists and 4 other dental auxiliaries serve this population of about 800 000 people). More than a third reported only visiting the hospital or clinic when their situation got worse (e.g. development of dento-alveolar abscesses, Ludwig's angina). Some TH do refer patients because they are aware of their limitations [41,42].

Proximity of the health care professional is a determinant in oral care delivery. In the Bui Division, primary oral health care is underdeveloped with poor access to basic oral health care. Poor access to oral health facilities limits patient's choices and hence patients prefer to patronize a TH rather than having to travel for more than 4 hours to see an oral health care worker.

## Conclusion

The majority of Cameroonians depend on traditional medicines for their health needs with about 7% of the average household health budget is spent on traditional medicines irrespective of the earning of its inhabitants. All TH operate full-time and greatly out-number the oral health care providers.

Most people still rely on TH because their treatment is affordable and TH share their patient's culture, beliefs and values and understand their expectations of health care. Hence they are generally more accessible and acceptable as health care providers. Their methods of treatment are effective and less invasive in certain cases, as they make use of local herbs and medicinal plants, though there are sometimes hazards associated with their treatments.

In view of the fact that factors affecting the oral health seeking behavior of patients in this region cannot be easily removed in a short term, a multisectorial-population based primary health care approach may be an option to break down some of these barriers. This should be based on an empowerment model which will integrate basic oral health care to all aspects of health care at PHC level equip TH with tools that that can assist them with the diagnosis and recording of oral disease, with appropriate referrals. Because TH have close contact with the community, simple basic dental practice applications like the Basic Package of Oral Care (BPOC) can be inculcated into their practice. They may also play a role in educating the community on the use of fluoride in the prevention of dental caries (AFT), restoring teeth using the atraumatic restorative (ART) and minimal invasive (MIT) techniques and carrying out emergency care by simple extractions.

From the findings of the present study, we can conclude that TH play a vital role in the health seeking behaviors of the community. Improving their knowledge and cooperation with oral health workers will serve to reduce inequalities and improve the standard of oral health care. TH can form a bridge linking the community to oral health care providers; can serve as a valuable tool for population-based health prevention and promotion approaches in achieving health for all. Oral health workers in this region have to increase cooperation and collaboration with TH. Therefore, traditional healers could be trained as counselors and educators to disseminate oral health information and carry out minor prevention practices within their communities. A follow-up study in collaboration with plant scientists to investigate the plants being used by TH for healing oral problems will be useful.

## Competing interests

The authors declare that they have no competing interests.

## Authors' contributions

MA contributed to the design of the study as well as acquisition of data, its analysis and interpretation and was involved in the drafting of the manuscript. SN made substantial contributions to the conception and design and in the drafting and revision of the manuscript. All authors read and approved the final manuscript.
